# Inhalation of Hydrogen Attenuates Progression of Chronic Heart Failure via Suppression of Oxidative Stress and P53 Related to Apoptosis Pathway in Rats

**DOI:** 10.3389/fphys.2018.01026

**Published:** 2018-07-31

**Authors:** Jing Chi, Zizhuo Li, Xiaojian Hong, Tong Zhao, Yueyue Bie, Wen Zhang, Jiaxing Yang, Ziming Feng, Zhouqi Yu, Qiannan Xu, Luqi Zhao, Weifan Liu, Yunan Gao, Hongxiao Yang, Jiemei Yang, Jiaren Liu, Wei Yang

**Affiliations:** ^1^Department of Cardiology, The First Affiliated Hospital of Harbin Medical University, Harbin, China; ^2^Department of Abdominal Ultrasonography, The First Affiliated Hospital of Harbin Medical University, Harbin, China; ^3^Department of Cardiology, The Fourth Affiliated Hospital of Harbin Medical University, Harbin, China; ^4^Department of Clinical Lab, The Fourth Affiliated Hospital of Harbin Medical University, Harbin, China

**Keywords:** hydrogen, chronic heart failure, oxidative stress, apoptosis, p53

## Abstract

**Background:** Continuous damage from oxidative stress and apoptosis are the important mechanisms that facilitate chronic heart failure (CHF). Molecular hydrogen (H_2_) has potentiality in the aspects of anti-oxidation. The objectives of this study were to investigate the possible mechanism of H_2_ inhalation in delaying the progress of CHF.

**Methods and Results:** A total of 60 Sprague-Dawley (SD) rats were randomly divided into four groups: Sham, Sham treated with H_2_, CHF and CHF treated with H_2_. Rats from CHF and CHF treated with H_2_ groups were injected isoprenaline subcutaneously to establish the rat CHF model. One month later, the rat with CHF was identified by the echocardiography. After inhalation of H_2_, cardiac function was improved vs. CHF (*p* < 0.05), whereas oxidative stress damage and apoptosis were significantly attenuated (*p* < 0.05). In this study, the mild oxidative stress was induced in primary cardiomyocytes of rats, and H_2_ treatments significantly reduced oxidative stress damage and apoptosis in cardiomyocytes (*p* < 0.05 or *p* < 0.01). Finally, as a pivotal transcription factor in reactive oxygen species (ROS)-apoptosis signaling pathway, the expression and phosphorylation of p53 were significantly reduced by H_2_ treatment in this rat model and H9c2 cells (*p* < 0.05 or *p* < 0.01).

**Conclusion:** As a safe antioxidant, molecular hydrogen mitigates the progression of CHF via inhibiting apoptosis modulated by p53. Therefore, from the translational point of view and speculation, H_2_ is equipped with potential therapeutic application as a novel antioxidant in protecting CHF in the future.

## Introduction

Because chronic heart failure (CHF), the end-stage of various heart diseases, continues to cause substantial morbidity and mortality, the ideally treatment for CHF needs to improve. CHF is a complex syndrome characterized by defecting bioenergetics, altering signal transduction pathways, and abnormal calcium homeostasis. In addition, apoptosis serves as another mechanism for the aggravation of CHF (Aubert et al., 2013; Li J. et al., 2014; Gorski et al., 2015). Acute oxidative stress causes severe injury to tissues (Kotur-Stevuljevic et al., 2015), and continuous oxidative stress is one of the reasons of abundant chronic diseases, tumor and senility (Sundar et al., 2013; Granados-Principal et al., 2014; Miller and Sadeh, 2014). A majority of intracellular reactive oxygen species (ROS) are by-products of mitochondrial metabolism. Bioenergetic activity and mitochondrial dysfunctions result in the generation of excess amounts of oxidant stress and further enhance cardiomyocytes apoptosis (Okonko and Shah, 2015).

C-Jun N-terminal kinase (JNK)-P53 is an important signaling pathway in oxidative stress. It is activated by ROS and then mediates apoptosis which related to the Bax-Bcl-2 complex in colistin-treated PC-12 cells (Lu et al., 2017). P53, as a gene of tumor suppression, plays an important role mainly through transcription of target genes referring to some processes, such as apoptosis, redox reaction, and cell cycle (Krifka et al., 2013). P53 is able to cause cell apoptosis by activating Bax directly or activate puma to up-regulating Bax and Bak indirectly (Zhang et al., 2016). However, studies on p53 of cardiomyocytes in the failing heart are rare.

Molecular hydrogen (H_2_) has many potential therapeutic applications as a novel antioxidant. In 2007, the first article reported H_2_ keeps tissues and cells safe from oxidative stress injury by eliminating ROS. As regulatory signaling molecules, ROS plays an important role in a mass of signal transduction cascades and also modulates bioprocess—apoptosis, for instance (Ohsawa et al., 2007). From then on, accumulating evidence has shown the protective action of H_2_ in various disease models (Guo et al., 2015; Kishimoto et al., 2015), including the infarct size of heart and brain by reducing ischemia-reperfusion injury without changing hemodynamic index, and protection against multiple-organ damage arising from generalized inflammation (Hayashida et al., 2008; Zhai et al., 2013; Miller and Sadeh, 2014). Some primary clinical trials also were performed in recently years (Ostojic et al., 2014; Yuan and Shen, 2016). About cardiovascular diseases, most H_2_-delivery ways were hydrogen-rich saline injection, which is not convenient. However, there was not reported about inhalation administration of H_2_ in the cardiovascular system.

Previous studies have demonstrated that hydrogen-containing saline treatment ameliorates the deterioration of doxorubicin-triggered heart failure in rats, which was likely mediated by its anti-autophagic or anti-apoptotic biological effects (Wu et al., 2014; Gao et al., 2016, 2017). However, the molecular mechanism underlying H_2_-induced anti-apoptosis effect in CHF was still unknown. In order to further clarify the aforementioned mechanism, the aims of this study were to determine to use much more convenient therapy—inhalation of H_2_, to completely investigate changes in the cardiac function and structure. Furthermore, signaling pathways mediated H_2_-induced inhibition of apoptosis in cardiomyocytes were explored, especially effects of p53 in rats with CHF. Our results provided new insights into molecular mechanisms that H_2_ gas inhibited apoptosis in CHF both *in vivo* and *in vitro*. We expect that in the near future H_2_ will be used clinically through inhalation as a novel antioxidant to protect against deterioration in CHF patients.

## Materials and Methods

### Animals

**FIGURE 1:**
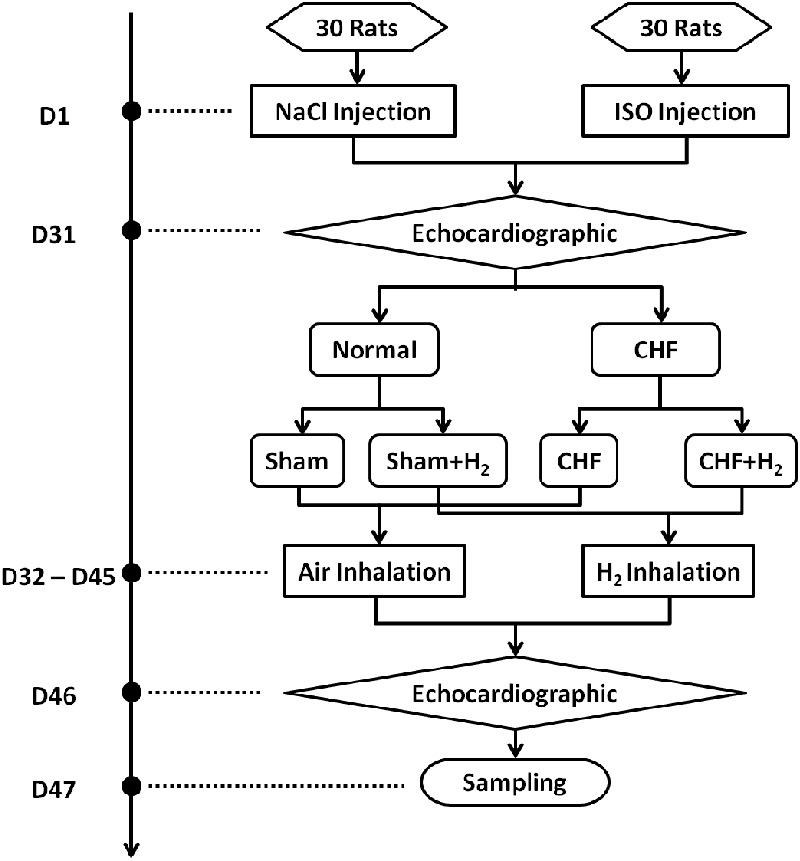
The time course of the study on each animal. The protocols and interventions on each animal and when they were performed were indicated.

Sixty Sprague-Dawley (SD) rats (male, 180–220 g; 6–7 weeks) were purchased from Liaoning Changsheng Biotechnology Co. Ltd. (China). The animals were fed in the room with natural light. The rats had food and water *ad libitum* and were food-deprived for 12 h before surgery. The study was conducted in accordance with the “Guidelines for the Care and Use of Laboratory Animals” (Fletcher and Crossgrove, 2011). All protocols were approved by the Animal Ethics Screening Committee (The First Affiliated Hospital of Harbin Medical University, China, clearance number 2017006). Rats were separated into four groups at random (15/group): Sham, rats treated with H_2_, CHF and CHF treated with H_2_ (**Figure 1**).

### Establishment of Heart Failure Model

Large doses of isoprenaline lead to injury of myocardium. The damaged heart gradually progress to heart failure. Isoprenaline was diluted in a sterile environment in saline immediately before the injection (Grimm et al., 1998). To confirm the most effective dose which could cause heart failure and along with an acceptable rate of survivors, three different doses of isoprenaline (I5627, Sigma-Aldrich, United States) were tested: 85, 170, and 340 mg/kg body weight (b.w.) (*n* = 10 for each group) were given subcutaneously once a day for two consecutive days. One month later, the 340 mg/kg was turned up to be the most ideal dose to beat the targets (**Table 1**). CHF was defined as LVEDD > 6.0 mm and EF < 60% by echocardiography (Wu et al., 2007). Animals that did not meet the criteria of CHF were excluded from the study. The rats without heart failure were executed in the manner of spinal dislocation.

**Table 1:** CHF rate and survival rate at each dose of ISO.

	85 mg/kg (*n* = 10)	170 mg/kg (*n* = 10)	340 mg/kg (*n* = 10)
ISO induced death (rats)	4	5	2
Anesthesia induced death (rats)	2	1	1
Survival (rats)	4	4	7
Survival rate (%)	40	40	70
CHF (rats)	1	2	6
CHF rate (%)	10	20	60

### Echocardiography

Echocardiography was performed using an animal-specific Doppler ultrasound system (S12-4, Philips CX50, Holland). Animals were intraperitoneally anesthetized by thiobutabarbital sodium (T133, Sigma-Aldrich, United States; 100 mg/kg b.w.), and M-mode images were recorded when the heart rates of the rats were maintained at 350–450 beats per minute. Left ventricular end-diastolic dimension (LVEDD), left ventricular end-systolic dimension (LVESD), right ventricle (RV), left atrium (LA), right atrium vertical diameter (RAVD), right atrium transverse diameter (RATD), interventricular septum (IVS), left ventricular posterior wall (LVPW), thickness of RV, E peak/mitral A peak (E/A), ejection fraction (EF), and fractional shortening (FS) were measured in this study. Three experienced technicians executed the measurement which was averaged for five consecutive cardiac cycles. The technicians were unaware of the identities of the grouping of animals.

### Hydrogen Treatment of Rats

**FIGURE 2:**
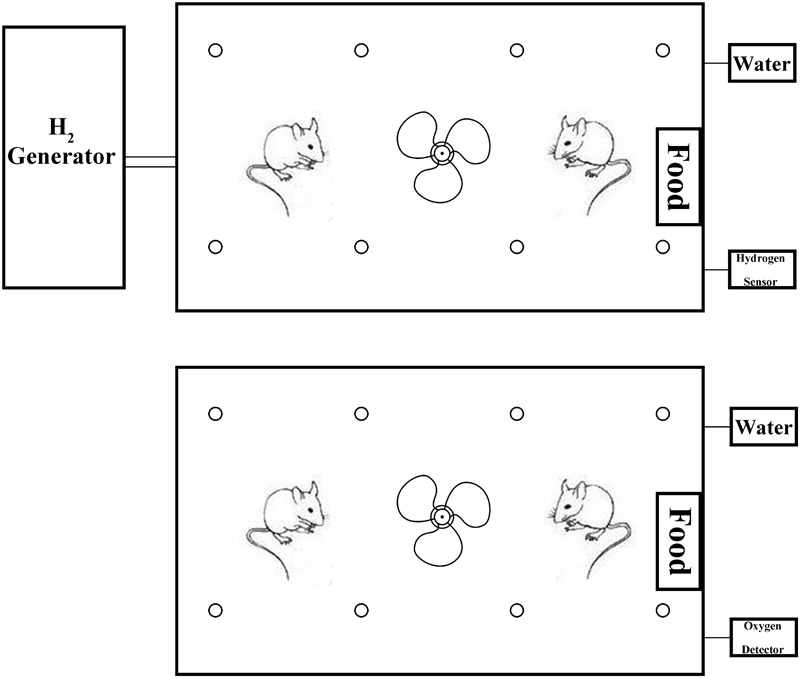
The H_2_ and air intake device for the rats. The fan in the ventilated box contributes to flowing of the air or H_2_. The box contains food, water, and bedding. Therefore, the rats could live in the box for a long time.

Hydrogen was prepared by the hydrogen generator (HA-300, SCDEALL, China; Output pressure: 0.4 MPa, Flow rate: 80 ml/min). Hydrogen was fed to a ventilated box which contained rats (ROX, 44 cm × 74 cm × 23 cm, Tenma, Japan). The concentration of hydrogen was maintained at 2% monitored by hydrogen sensor (SDH-B101, Honeywell, United States). Another ventilated box containing rats was filled with air. For successive 14 days, rats were treated in the two boxes for 12 h everyday (**Figure 2**).

### Hydrogen and Oxygen Measurement

H_2_ and oxygen (O_2_) dissolved in the solution and blood was measured with the method described before (Ohsawa et al., 2007). H_2_ and O_2_ concentrations were determined by hydrogen sensor (SDH-B101, Honeywell, United States) and oxygen detector (AR8100, Smart Sensor, Hong Kong, China).

### Histology

Heart tissues were fixed with 4% paraformaldehyde and imbedded in paraffin; At the left ventricle (LV) papillary muscle level, tissue sections (4-μm thickness) of the hearts were sliced and stained with Masson trichrome, terminal-deoxynucleoitidyl transferase mediated nick end labeling (TUNEL, In Situ Cell Death Detection Kit, POD, 11684817910, Roche, Switzerland), and immunohistochemical methods using anti-p53 (OP33, Millipore, United States) and 8-OHdG (ab10802, Abcam, United States). The immunostaining of p53 was performed according to the immunohistochemical protocol from Millipore using p53 antibody (1:100 dilution). Digital photomicrographs were taken at magnification of 200×, and five random fields from each section were selected and analyzed with unawareness of animal groups. Size of fibrosis, apoptosis cardiomyocytes, p53 and 8-OHdG positive cells were measured in five sections from each heart, and the mean value was calculated.

### Measurement of Brain Natriuretic Peptide (BNP)

Blood samples from abdominal aorta of all survived rats were collected into heparin-containing tubes, centrifuged at 3000 × *g*, 4°C for 15 min. Plasma was collected and tested within 2 h. The plasma concentrations of BNP were measured with ELISA kits according the manufacturers’ instructions (H166, Nanjing Jiancheng Bioengineering Institute, China). The experiment mentioned above was replicated for three times and intra-assay coefficients of variation were <10% for all assays.

### Cell Culture

Neonatal rats (0–3 days) were acquired from the Laboratory Animal Center of the Second Affiliated Hospital of Harbin Medical University (Harbin, China). Cardiomyocytes were isolated and cultured in the medium which contained Dulbecco’s modified eagle medium (DMEM, SH30022.01, GE, United States), 10% fetal bovine serum (FBS) and 1% penicillin–streptomycin, pH 7.1, then maintained at 37°C in 95% air and 5% CO_2_. Brdu (B5002, Sigma-Aldrich, United States) was added to the media for inhibiting the growth of fibroblasts. Cardiomyocytes were tested using the Anti-Cardiac Troponin T (TnT) antibody (ab92546, Abcam, United States) by immunofluorescence. The densities of both cardiomyocytes and fibroblasts reached 80%, respectively. Prior to all experiments, cells were serum-starved for 16 h. Cells with 70–90% confluence were used for experiments. Menadione (Vitamin K_3_, VK_3_; M5626, Sigma-Aldrich, United States) was added into the medium to make oxidative stress model, and final concentration was maintained at 10 μM. Then cells were treated with hydrogen for 2 h, 4 h, 6 h, 8 h, 12 h, 24 h, and 72 h. With optical microscope, cells were observed when treated with hydrogen until all died (72 h); cells died partially were treated with hydrogen for 24 h, and cells in good conditions were treated with hydrogen for 2 h, 4 h, 6 h, 8 h, and 12 h. Therefore, treatment of hydrogen for 8 h were chosen to conduct subsequent experiments.

### Cell Death Assay

The death of cardiomyocyte was evaluated by the staining of trypan blue (T6146, Sigma-Aldrich, United States). Cells (5.0 × 10^5^) were incubated with 0.4% trypan blue solution for 10 min at room temperature. The blue dead cells and all the cells were counted, respectively. The percentage of dead cells was calculated (%). Each experiment was performed in triplicate.

### Hydrogen Treatment of Cultured Cells

Hydrogen treatment of cultured cells was carried out as described before (Ohsawa et al., 2007). The control group was DMEM, 10% FBS and 1% penicillin–streptomycin, with mixed gas (75% N_2_, 20% O_2_, and 5% CO_2_).

### Overexpression of P53 in H9c2 Cells

H9c2 cells from rat embryonic ventricular myocardium were acquired from Cell Bank of Chinese Academy of Sciences [H9c2 (1-2), China]. Full-length p53 was obtained from liver tissue of rat, then subcloned into plasmid for lentivirus expression vector (pLVX, Clontech, United States) between EcoRI and BamHI sites. Lentivirus for overexpressing rat P53 was produced in 293T cells. The sense and antisense primers for rat p53 were as follows: 5′-TATGTCTAAGGGACCTGCGGTTGGCATTGATCTTG-3′ and 5′-GTGCCAAGATCAATGCCAACCGCAGGTCCCGGAG ACA-3′. H9c2 cells from matched group were infected with pLVX. Lentivirus with Enhanced Green Fluorescent Protein (EGFP) was applied to infect H9c2 cells to confirm the optimum concentration and to assess the efficacy of infection. H9c2 cells were infected with 0, 5, 10, 20, or 30 μl/ml lentivirus for 6 h. The day after infection, the cells were selected with 0, 0.1, 0.2, 0.4, 0.8, 1.0, and 1.5 μg/ml puromycin. The cells were visualized by fluorescence microscopy at 24 h post infection (Vert. A1, HBO 50, ZEISS, Germany). After puromycin screening for 48 h, EGFP-stable clones were screened. The percentages of cells with EGFP were determined from four separate visual fields. The optimal doses were 20 μl/ml lentivirus and 0.8 μg/ml puromycin. The process related to the lentivirus were handled following the guideline of biosafety level 2. The selected cells were starved before VK_3_ and H_2_ treatment for 16 h. The expression of p53, Bax and cleaved caspase-3 were determined by Western blotting.

### Inhibition of P53 in H9c2 Cells

For inhibition of p53 in response to H_2_, H9c2 cells were treated with Pifithrin-α (PFTα, S1816, Beyotime, China), which was a p53 inhibitor that dissolved in dimethyl sulfoxide (DMSO). Chu et al. (2006) have reported that expression of p53 in H9c2 cells was inhibited after the cells were treated with 20 μM PFTα and doxorubicin for 8 h. However, we observed that after treatment with 20 μM PFTα and 10 μM VK_3_, H9c2 cells all died 2 days later. Therefore, the final concentration of PFTα was 10 μM in the DMEM in this study. DMSO as a control was given the same volume to PFTα to H9c2 cells. After 16 h, the culture medium was changed to DMEM with 1% FBS, 1% penicillin–streptomycin and 10 μM PFTα and then the treatments of VK_3_ and H_2_ were continued.

### TUNEL Staining

The TUNEL assay was carried out to label the 3′-end of fragmented DNA in tissue slices or cardiomyocytes in accordance with the manufacturer’s directions (Roche, Switzerland) and stained with DAB kit (ZLI-9018, ZSGB-BIO, China). The TUNEL-positive area was expressed as the percentage of the total area. Finally, slides were observed by a light microscope (DP72, Olympus Co., Japan) and fluorescence microscope, and quantitative statistical analysis was performed with the Medical Image Analysis System (Motic Med 6.0, China).

### Measurement of Malondialdehyde (MDA)

Myocardial tissues from survived rats or neonatal rat cardiomyocytes were homogenized in ice-cold phosphate buffer saline. MDA, a marker of lipid peroxidation, was detected with a commercial kit (KGT003, KeyGEN Biotech. Co. Ltd., China). The MDA concentration was represented as the ratio of MDA content and the concentration of proteins which was tested with bicinchoninic acid (BCA) method (P0012, Beyotime Biotechnology, China). The experiment mentioned above was triplicated.

### Western Blotting

Protein from heart tissues and cardiomyocytes were fractionated by sodium dodecyl sulfate-polyacrylamide gel electrophoresis (SDS-PAGE) and then electrotransferred to poly vinylidene fluoride (PVDF) membranes (Roche, Switzerland). The blotted membranes were incubated with antibodies against cleaved-caspase-3 (#9664, Cell Signaling Technology, United States), p53 (#2524, Cell Signaling Technology), phospho-p53 (#12571, Cell Signaling Technology), Bax (ab182733, Abcam, United States), bcl-2 (ab59348, Abcam) or glyceraldehyde-3-phosphate dehydrogenase (GAPDH, AB2000, Abways, China). Immunoreactivity was detected using an enhanced chemiluminescence reaction system (Universal Hood II, Bio-Rad, United States). Each experiment was performed in triplicate.

### Statistical Analysis

The data were expressed as mean ± SD or mean ± SE. The normal frequency distribution was analyzed with Kolmogorov–Smirnov test and the homogeneity of variances was analyzed with Levene statistics. The multiple group comparison was carried out with one-way analysis of variance (ANOVA) and least significant differences *t*-test. The tests were two-sided and *p* < 0.05 was regarded as statistical significance, beta-error was 0.2. A rank sum test was used when there was heterogeneity of variance or abnormal frequency, and values of *p* < 0.0083 (α′ = 0.0083) were considered as statistically significant (α′ = α/N, *N* = C^2^_n_ = *n* × (*n* - 1)/2, *n* = 4, α = 0.05). All of statistical analyses were carried out with Statistical Product and Service Solutions (SPSS) 24.0 software (IBM, New York, NY, United States) (Chi et al., 2014).

## Results

### H_2_ Improved Cardiac Function in Rats

**FIGURE 3:**
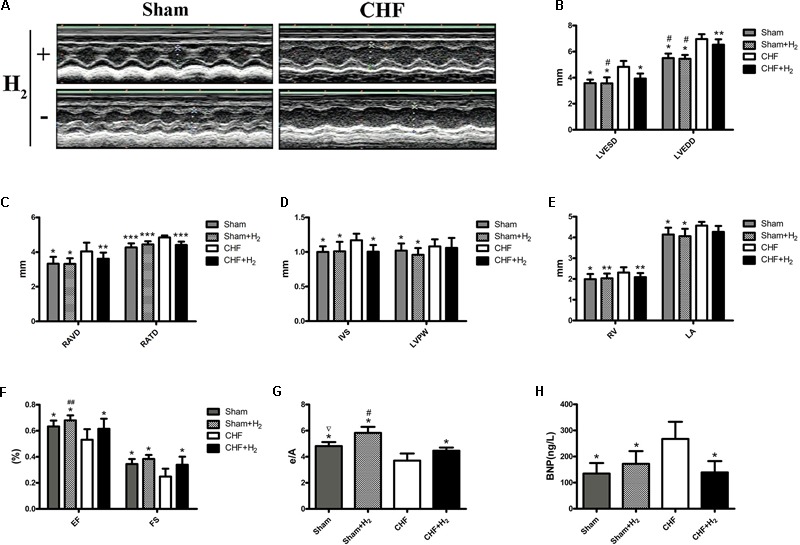
Cardiac structure and function. **(A)** One month post-ISO injection, the rats were treated with H_2_ for 14 days. Cardiac structure and function were measured with M-mode echocardiography. Representative M-mode echocardiographic photographs of LV are shown. **(B–G)** LVESD, LVEDD, RAVD, RATD, IVS, LVPW, RV, LA, EF, FS, and E/A values are shown (*n* = 9–11). **(H)** BNP level in serum (*n* = 10). The columns indicate the mean, and the bars indicate the SD. ^∗^*p* < 0.01 vs. CHF; ^∗∗^*p* < 0.05 vs. CHF; ^∗∗∗^*p* < 0.0083 vs. CHF; ^#^*p* < 0.01 vs. CHF+H_2_; ^##^*p* < 0.05 vs. CHF+H_2_; ^∇^*p* < 0.01 vs. Sham+H_2_.

**FIGURE 4:**
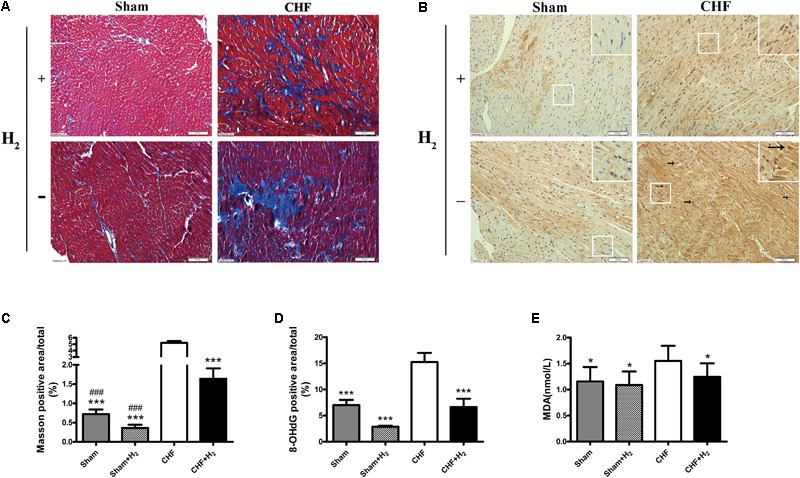
Left ventricular remodeling size and oxidative stress damage in myocardial tissue after inhalation of H_2_ and air. **(A)** Representative photomicrographs of Masson’s trichrome-stained heart sections are shown (magnification of 200×). Blue area represents fibrosis. Scale bars = 50 μm. **(B)** Representative photomicrographs of immunohistochemistry about 8-OHdG (brown nuclei, arrows) are shown (magnification of 200×). Scale bars = 50 μm. Inset: Magnified region illustrating 8-OHdG negative cardiomyocytes (blue nucleus) and positive ones (brown nucleus). **(C)** Left ventricular remodeling area was determined by measuring the area of fibrosis and was expressed as a percentage of the whole LV area (*n* = 7–9). **(D)** 8-OHdG was determined by measuring the positive area of brown nucleus and was expressed as a percentage of the whole section area (*n* = 8–10). **(E)** MDA levels in myocardial tissue are shown (*n* = 12–14). The columns indicate the mean, and the bars indicate the SD (LVEDP, LVESP, and MDA) or SE (+dp/dt, –dp/dt, Masson, and 8-OHdG). ^∗^*p* < 0.01 vs. CHF; ^∗∗∗^*p* < 0.0083 vs. CHF; ^###^*p* < 0.0083 vs. CHF+H_2_.

To elucidate the role of H_2_ in CHF, myocardial injury was induced using isoprenaline in rats. One month later, 12–14 rats/group subject to the echocardiography, and 8–12 rats/group developed CHF. The rats with CHF were treated with H_2_ for 14 days. After treatment, cardiac function of rats was reexamined with echocardiography (**Figure 3A**). The LVESD, LVEDD, RAVD, RATD, IVS, and RV dramatically reduced when compared to the CHF group, however, there were no significant differences between CHF and CHF with H_2_ group in LVPW and left atrium (LA). In addition, LVEDD of the treatment group was still greater than matched group (**Figures 3B–E**). Meanwhile, EF, FS, and mitral E/A in rats with CHF were much lower than those in the control group. Besides, E/A in normal rats increased significantly after treating with H_2_ in comparison with untreated normal group (**Figures 3F,G**). Moreover, BNP, as an indicator of CHF in serum, showed similar results to the results from the echocardiography (**Figure 3H**). Furthermore, myocardial fibrosis was reduced after inhalation of H_2_ (**Figures 4A,B**). These results collectively suggest that H_2_ may prevent the development of CHF and protect the cardiac function in rats.

### H_2_ Released Oxidative Stress Injury in CHF

H_2_-mediated molecular changes of CHF was detected, by incubating heart slices with the antibody of 8-OHdG to evaluate the level of nucleic acid oxidation. As the oxidative marker, 8-OHdG staining was substantially reduced after H_2_-treatment in both normal and CHF rats (**Figures 4C,D**). Furthermore, the level of MDA was examined in myocardial tissues, which reflected the degree of lipid peroxidation. As expected, the results showed that the level of MDA was significantly decreased after treated with H_2_ when compared to untreated rats which subjected to CHF (**Figure 4E**). All in all, these results indicated that H_2_ is capable of attenuating oxidative stress markedly and decreasing damage to the myocardium.

### H_2_ Decreased Apoptosis in CHF

**FIGURE 5:**
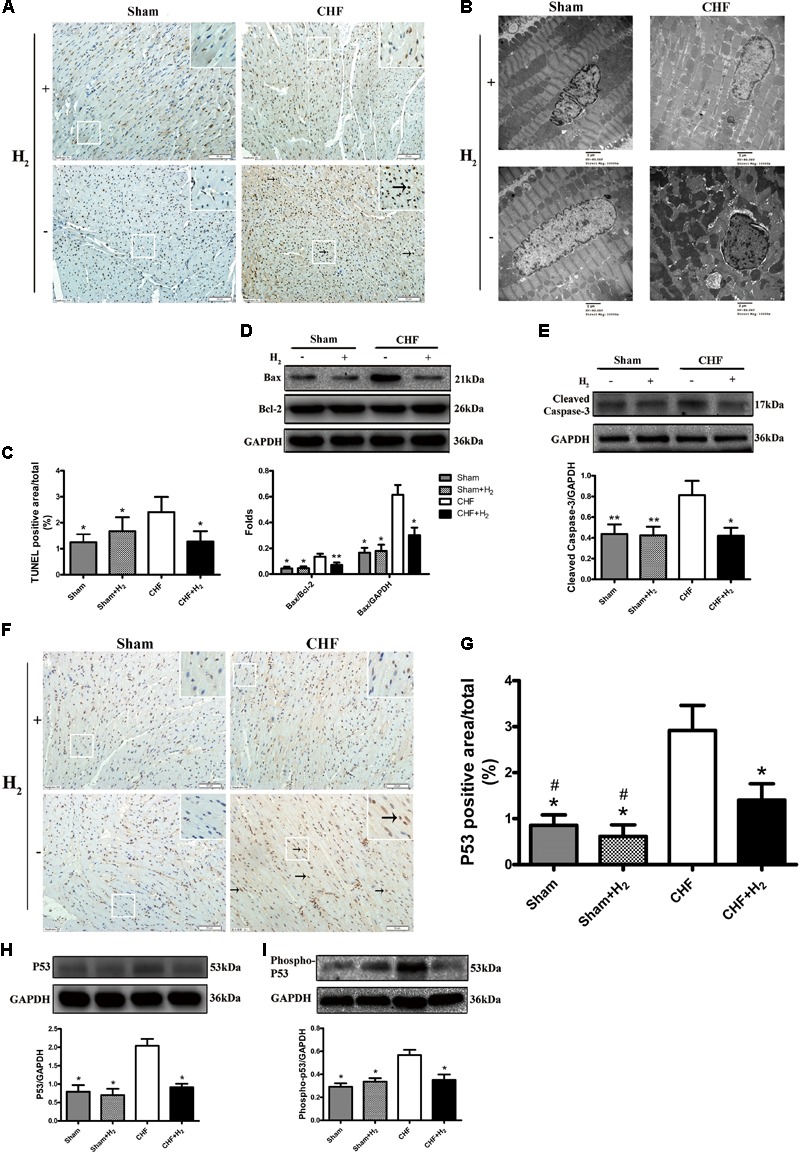
Effects of H_2_ on apoptosis and cardiac p53 protein expression in CHF. **(A)** Apoptosis was detected by TUNEL assay (brown nuclei, arrows). Representative photomicrographs are shown (magnification of 200×). Scale bar = 50 μm. Inset: Magnified region illustrating non-apoptotic cardiomyocytes (blue nucleus) and apoptotic ones (brown nucleus). **(B)** Nuclei of cardiomyocyte was evaluated by observation under electronic microscopy (magnification of 10,000×). Scale bar = 2 μm. **(C)** TUNEL staining was quantified as the percentage of apoptotic cardiomyocytes of the whole section area (*n* = 8–9). **(D,E)** Confirmation by Western blot analyses for the expressions of cardiac Bax and cleaved-caspase-3. The ratios of Bax and bcl-2 are shown (*n* = 8–10). **(F)** Representative photomicrographs of immunohistochemistry about p53 (brown nuclei, arrows) are shown (magnification of 200×). Scale bar = 50 μm. Inset: Magnified region illustrating p53 negative cardiomyocytes (blue nucleus) and positive ones (brown nucleus). **(G)** P53 was determined by measuring the positive area and was expressed as a percentage of the whole section area (*n* = 8–9). **(H,I)** Confirmation by Western blot analyses for the expression of cardiac p53 and phospho-p53. The ratios of phospho-p53 and p53 are shown (*n* = 10). Representative photographs are shown. GAPDH was used as a loading control. Expressions of cleaved-caspase-3, Bax, p53 and phospho-p53 were, respectively, quantified as folds of GAPDH. The columns indicate the mean, and the bars indicate the SD (TUNEL and p53 staining) or SE (Western blotting). ^∗^*p* < 0.01 vs. CHF; ^∗∗^*p* < 0.05 vs. CHF; ^#^*p* < 0.01 vs. CHF+H_2_.

The effects of H_2_ modulation on apoptosis were also determined in rats with CHF. After treatment with H_2_, the number of apoptotic cells was significantly reduced in cardiac tissues (**Figures 5A,C**). Meanwhile, karyopyknosis was mitigated after the rats were treated with H_2_ (**Figure 5B**). Moreover, the expression of Bax which promotes apoptosis was attenuated by H_2_ (**Figure 5D**). Likewise, cleaved-caspase-3 as a marker of executing apoptosis was significantly reduced after H_2_ treatment (**Figure 5E**). These results collectively suggest that H_2_ protects heart against exhaustion partly by reducing apoptosis of cardiomyocytes.

### P53 Plays a Vital Role in the Effects of H_2_ on CHF

**FIGURE 6:**
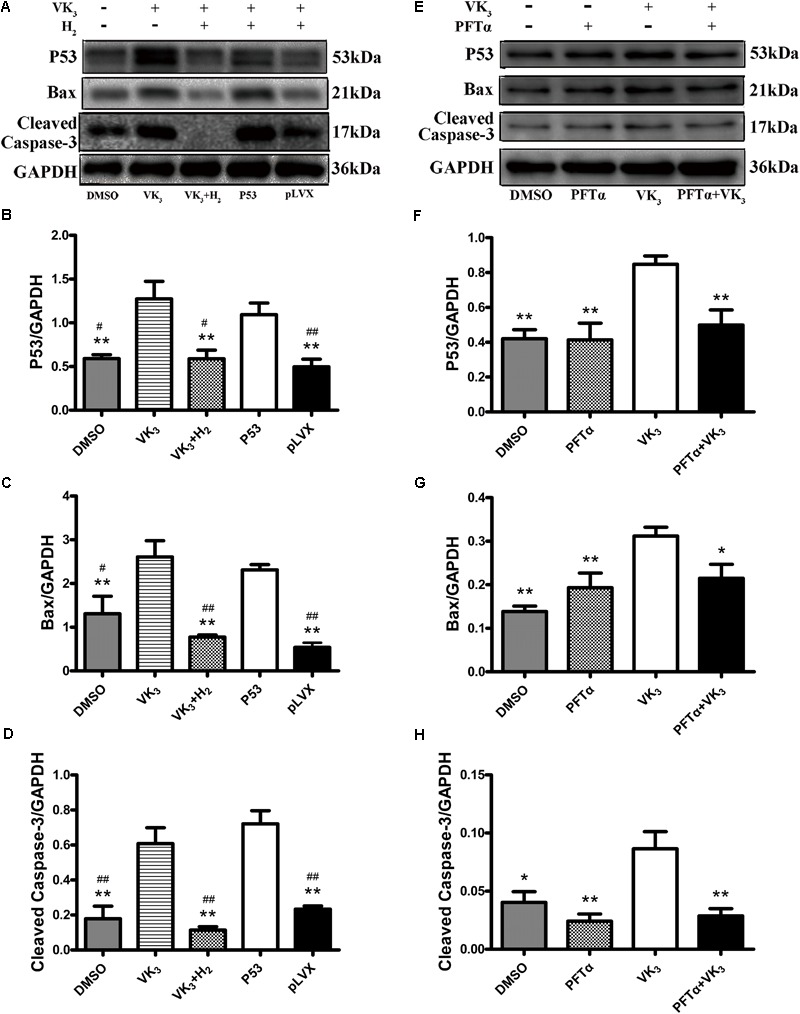
Analysis for effects of H_2_ on the expression of p53 in H9c2 cells. **(A–D)** Overexpression of p53 in H9c2 cells. In the groups of P53 and pLVX, H9c2 cells were infected with p53-lentivirus or pLVX and were selected for 72 h. Then the cells were treated with 10 μM VK_3_ and 75% H_2_ for 8 h. In the groups of DMSO, VK_3_ and VK_3_+H_2_, the cells were treated as the primary cardiomyocytes. **(E–H)** Inhibition of p53 in H9c2 cells. H9c2 cells were treated with 10 μM PFTα or equivoluminal DMSO for 16 h (DMEM group and PFTα), and then treated with 10 μM VK3 for 8 h (VK3 group and PFTα+VK_3_ group). Western blot analysis was performed on 100 g cell lysates with antibodies against p53, Bax, or cleaved caspase-3. GAPDH was used as a loading control. Expressions of p53, Bax, and cleaved caspase-3 were, respectively, quantified as folds of GAPDH (*n* = 3–4). The columns indicate the mean, and the bars indicate the SE. ^∗^*p* < 0.05 vs. VK_3_; ^∗∗^*p* < 0.01 vs. VK_3_; ^#^*p* < 0.05 vs. P53; ^##^*p* < 0.01 vs. P53.

It is known that the tumor suppressor p53 regulates apoptotic process of cardiomyocyte and is profoundly involved in the development of CHF (Sun et al., 2014). Therefore, whether modulation of H_2_ could affect the expression and phosphorylation of p53 was further examined. The level of p53 protein was significantly increased in failing hearts and decreased in the H_2_-treated CHF (**Figures 5F,G**). Besides, the expression and phosphorylation levels of p53 in cardiomyocytes were significantly increased in rats with CHF and decreased after H_2_-treated CHF (**Figures 5H,I**). Meanwhile, as found in rat primary cardiomyocyte, the inhibitory effect of H_2_ on VK_3_ was also found in H9c2 cells, however, overexpression of p53 restrained this effect (**Figures 6A–D**). As found in rat primary cardiomyocyte, the expression of p53, Bax and cleaved-caspase-3 was increased by the stimulation of VK_3_ in H9c2 cells, however, inhibition of p53 restrained the stimulative effect (**Figures 6E–H**). Taken together, the results above illustrated that p53 plays a vital role in the modulation of H_2_ in CHF.

### H_2_ Reduced Apoptosis Caused by Oxidative Stress in Primary Cardiomyocytes

**FIGURE 7:**
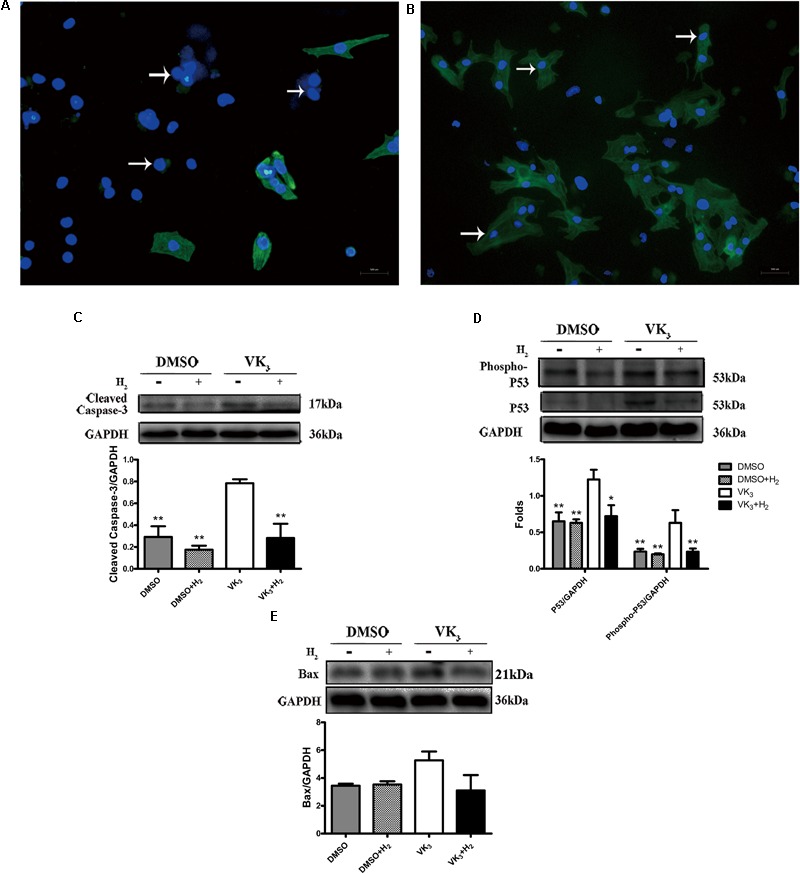
Analysis of effects of H_2_ on primary cardiomyocytes with oxidative stress. **(A,B)** Identification of primary cardiomyocytes. Representative photomicrographs of fluorescence are shown. Myofilaments were marked with the green fluorescence, representing the expression of TnT (**B**, arrows) which was negative in fibroblasts (**A**, arrows) (magnification of 100×). Scale bar = 500 μm. **(C–E)** Confirmation by Western blot analyses for the expression of cardiac cleaved caspase-3, phospho-p53, p53, and Bax in primary cardiomyocytes. Representative photographs are shown. GAPDH was used as a loading control. Expressions of cleaved-caspase-3, phospho-p53, p53 and Bax were, respectively, quantified as folds of GAPDH (*n* = 3–5). The columns indicate the mean, and the bars indicate the SD (MDA and cell viability) or SE (Western blotting). ^∗^*p* < 0.05 vs. VK_3_; ^∗∗^*p* < 0.01 vs. VK_3_; ^&&^*p* < 0.01 vs. VK_3_+H_2_.

**FIGURE 8:**
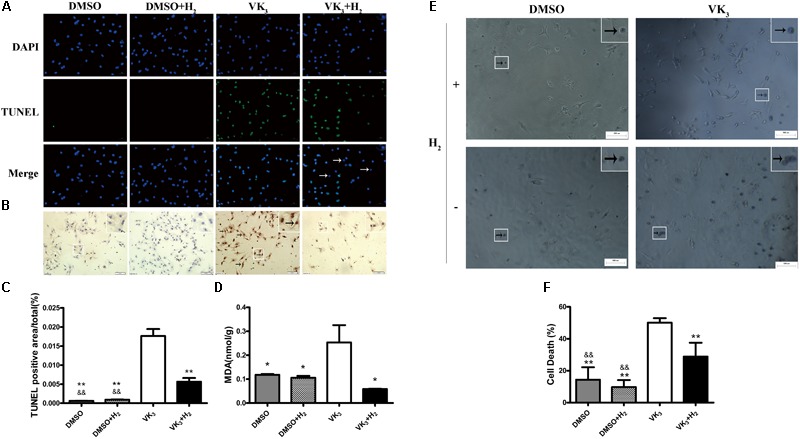
Cell death and oxidative stress analyses for primary cardiomyocytes. **(A)** Apoptosis was detected by TUNEL assay. Representative photomicrographs of fluorescence are shown. Green nuclei represented apoptosis. All of nuclei were stained with DAPI (blue). After merging, blue-green nuclei represented apoptosis. Arrows represented the blue nuclei that were not apoptosis (Magnification of 100×). Scale bar = 500 μm. **(B)** Representative photomicrographs of DAB staining are shown. Brown nuclei (arrows) represented apoptosis (magnification of 200×). Scale bar = 50μm. Inset: Magnified region illustrating non-apoptotic cardiomyocytes (blue nucleus) and apoptotic ones (brown nucleus). **(C)** TUNEL staining was quantified as the percentage of apoptotic cardiomyocytes (brown nuclei) of the whole section area (*n* = 3). **(D)** MDA levels in primary cardiomyocytes are shown (*n* = 3). **(E,F)** Cell death assay for primary cardiomyocytes (*n* = 5). Representative photomicrographs of trypan blue staining are shown. Blue cells (arrows) represented cell death (magnification of 100×). Scale bar = 500 μm. Inset: Magnified region illustrating dead cardiomyocytes (blue staining). The columns indicate the mean, and the bars indicate the SD. ^∗^*p* < 0.05 vs. VK_3_; ^∗∗^*p* < 0.01 vs. VK_3_; ^&&^*p* < 0.01 vs. VK_3_+H_2_.

Modulatory effects of molecular hydrogen on apoptosis were also determined in cultured primary cardiomyocytes that underwent oxidative stress injury (**Figures 7A,B**). The expression of cleaved-caspase-3, p53 and phosphor-p53 was down-regulated by H_2_ treatment after oxidative stress injury (**Figures 7C,D**), which was similar to the results from *in vivo*. However, Bax transcribed by p53 did not appear significant difference between oxidative stress and hydrogen treatment groups (**Figure 7E**). Furthermore, H_2_ treatment also attenuated the apoptosis caused by oxidative stress in cardiomyocytes (**Figures 8A–C**). In general, these results suggest that H_2_ could be a major player in protection of cardiomyocytes from oxidative stress-induced apoptosis.

### H_2_ Protected Primary Cardiomyocytes From Damage of Oxidative Stress

As is well-known that VK_3_ can be used for inhibiting complex I or complex III of the mitochondrial electron transport chain. Therefore, the leakage of electrons was accelerated and O_2_^-^ was produced. In order to test the protective effect of H_2_ on cardiomyocytes with oxidative stress, cardiomyocytes were exposed to 75% H_2_ for 8 h. After H_2_ treatment, the level of MDA in cardiomyocytes was examined in this study. The results showed that H_2_ could also reduce damage of oxidative stress in primary cardiomyocytes (**Figure 8D**). In addition, H_2_ also significantly reduced cell death of primary cardiomyocytes (**Figures 8E,F**).

## Discussion

### Key Findings of the Study

Our findings revealed that molecular hydrogen could markedly reduce oxidative stress and suppress myocardium injury *in vivo* and *in vitro*. Inhalation of H_2_ might prevent CHF from progressing and protect the cardiac function of rats partly by reducing apoptosis of cardiomyocyte. Meanwhile, H_2_ could also be confirmed effective effects on protection of cardiomyocytes from oxidative stress-induced apoptosis. Notably, the expression and phosphorylation of p53 play an important role in CHF when treated with hydrogen.

### Hydrogen Alleviated Continuous and Mild Oxidative Stress in Cardiomyocytes

Many studies from *in vitro* and *in vivo* have demonstrated that ROS are generated partly in the circulatory system, including the failing heart (Hafstad et al., 2013; Chouchani et al., 2014; Brown and Griendling, 2015). Continuous and mild oxidative stress may lead to injury in various organs and tissues (Jourdan et al., 2014; Li Y. et al., 2014). It has been shown that inhalation of H_2_ ameliorated oxidative myocardial injury during cardiopulmonary resuscitation and after returning of spontaneous circulation (Hayashida et al., 2012). Our previous study also indicated that the serum MDA level was decreased after injection of hydrogen-rich saline (Gao et al., 2017). In this study, the levels of MDA and 8-OHdG were examined in myocardial tissue, and found that inhalation of H_2_ ameliorated DNA damage of cardiomyocytes in rats with CHF. The experiments using cultured cardiomyocytes also showed that H_2_-treated myocardial lipid were more resistant to the VK_3_-induced oxidant stress. Interestingly, the level of 8-OHdG in cardiomyocytes also significantly decreased after inhalation of H_2_ in the normal rats, which needs a larger sample size to verify. Results from trypan blue assay indicated that H_2_ reduced cell death of cardiomyocytes, which has not been reported before. These findings suggest that inhalation of H_2_ alleviate the oxidative stress injury of cardiomyocytes both in neonatal rats and adult rats with CHF.

### Effects of Inhalation H_2_ on CHF Were Observed Completely for the First Time

Dysfunction of LV is related to oxidative stress in the myocardium and plasma of patients with heart failure (Maack et al., 2003). The treatment of CHF has been a worldwide puzzle on account of irreversible fibrosis, remodeling, and apoptosis. The anti-fibrosis effect of H_2_ has been verified in overload-induced cardiac dysfunction in rats recently (Yang et al., 2017). In this study, our findings found that inhalation of H_2_ may decrease the area of fibrosis in isoprenaline induced CHF. Moreover, our findings also confirmed that inhalation of H_2_ was able to improve cardiac function which was also verified by hemodynamic measurement. Furthermore, the safety of molecular hydrogen to cardiomyocytes has been confirmed as reported before (Tao et al., 2015). However, in our previous study, LVEDD in saline, doxorubicin and doxorubicin+hydrogen-rich saline groups was not found differences (Gao et al., 2017). Meanwhile, LVEDD, LVPW and LA from the treatment group did not decline to the baseline. Maybe a universal standard of CHF in rats should be established in the future. Additionally, the improvement of E/A indicates that the diastolic function may be improved by H_2_. Ventricular remodeling is a primary reason of deterioration of heart failure and death after myocardial damage (Ahmad et al., 2014). ROS may participate in the remodeling processes in various ways, such as activating matrix metalloproteinases (MMPs) that correlate with reconstitution of the extracellular matrix; functioning as signaling factors in the progress of vicarious hypertrophy; and resulting in cardiomyocytes death via apoptosis or other mechanisms (Liu et al., 2012; Yang et al., 2013; Li Y. et al., 2014; Zhang et al., 2014; Kessler et al., 2015). In addition, the result that E/A increased in normal rats compared with the matched group treated with H_2_ indicates that inhalation of H_2_ is not only harmless to myocardium but also helpful for diastolic function. Further clinical studies are needed to verify whether inhalation of H_2_ would be used in clinic.

### Suppression of Apoptosis Was Further Observed in Cardiomyocytes of CHF Treated With H_2_

Apoptosis of cardiomyocytes may be a cause and effect of CHF, and inhibition of this cell death process may contribute to the exploration of new treatment (Melman et al., 2015). ROS activate the JNK, p38 mitogen-activated protein kinases (MAPKs) and Akt and induces apoptosis (Hao et al., 2015; Santabárbara-Ruiz et al., 2015). In this study, we observed alleviated karyopyknosis by electron microscope and the number of apoptotic cardiomyocytes was decreased after treated with H_2_. In addition, studies of apoptosis-related protein *in vivo* and *in vitro* suggest that molecular hydrogen may inhibit apoptosis, which is in line with the studies before (Guo et al., 2015; Song et al., 2015; Gao et al., 2017). However, our present study revealed that after treated with VK_3_, the expression of Bax in primary cardiomyocytes was not suppressed by H_2_, but was suppressed in H9c2 cells. Therefore, a larger sample size is also needed to verify this result in the future. Moreover, Song et al. (2013) found that hydrogen-rich water protected activity of endothelial cells from apoptosis. In our study, a similar phenomenon on activity of cardiomyocytes was also found. In addition, our study also verified that molecular H_2_ decreased VK_3_ induced apoptosis in H9c2 cells. Therefore, we confirm that H_2_ may protect CHF from progressing in apoptosis pathway much more completely than before, especially in inhalation manner.

### P53 Mediated Apoptosis Pathway Intervened by H_2_ in Cardiomyocytes

As an important transcription factor, p53 plays a vital role in the signaling pathway of apoptosis induced by ROS (Li et al., 2015). P53 protein is not novel in apoptosis, however, it is an important factor in cardiomyocytes and is rarely studied in CHF in especial. Our present study revealed that molecular hydrogen reduced the expression and phosphorylation of p53 in cardiomyocytes for the first time. However, inhalation of H_2_ could not decrease the expression of p53 to the normal level. Our data suggest that molecular hydrogen may inhibit oxidative stress in CHF. Consequently, H_2_ might provide a suppressive signal to p53 leading to the attenuated p53 activity. The relative contributions of H_2_ in the ROS/p53/apoptosis signaling pathway and preferential protection of CHF were a meaningful subject to study. Hence, knockdown p53 expression or overexpressed p53 to observe the impact of H_2_ in protecting cardiomyocytes. When knockdown the expression of p53, H9c2 cells treated with VK_3_ could not increase the expressions of p53, Bax or cleaved-caspase-3, which was in line with the result of the study that doxorubicin induced apoptosis in H9c2 cells (Chu et al., 2006). When p53 was overexpressed in H9c2 cells, the protective effect of H_2_ on oxidative stress injury was weakened. Therefore, in H_2_ protecting process of cardiomyocytes, the modulating effect of p53 was firstly confirmed. We believe that the decreasing p53 protein modulated by H_2_ is important because it targets apoptosis in the treatment of CHF.

## Future Direction and Conclusion

**FIGURE 9:**
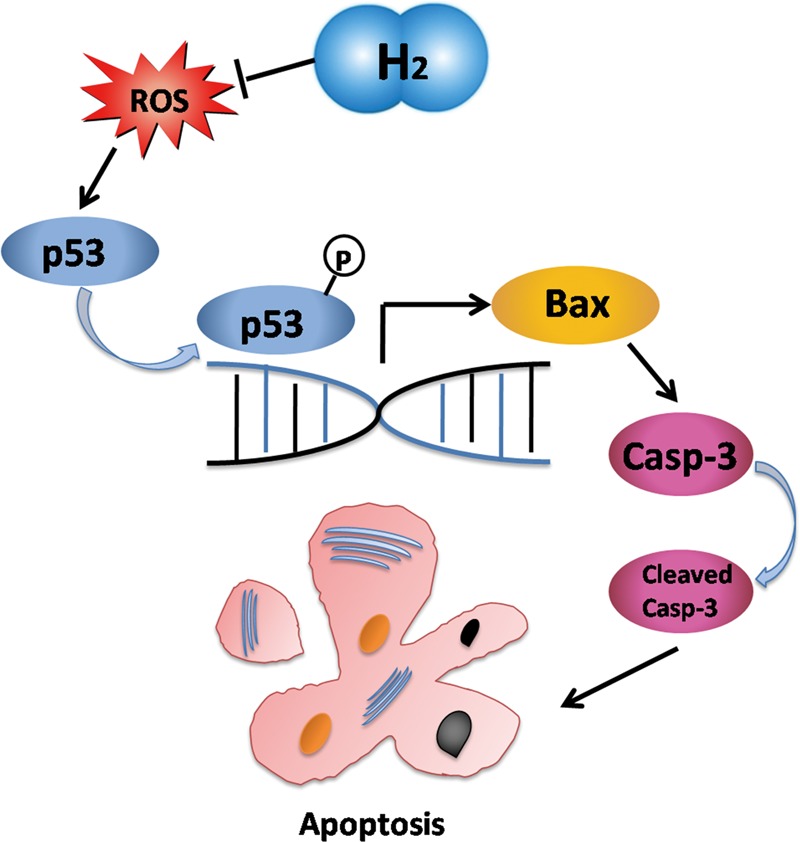
Schematic depicting the mechanisms by which molecular hydrogen mediates apoptosis. H_2_ eliminated oxygen free radicals which led p53 phosphorylation. As a transcription factor, phosphorylated p53 increased expression of Bax which increased expression of caspase-3. Cleaved-caspase-3 executed apoptosis of cardiomyocytes. Hence, apoptosis was suppressed by inhibition of p53 in response to molecular hydrogen.

Our study indicates that H_2_ protected cardiomyocytes and myocardial tissues of the failing heart against apoptosis by inhibiting oxidative stress. This process was carried out by inhibiting phosphorylation and transcription of p53 to Bax (**Figure 9**). Applying H_2_ in CHF may open up a new way for researching. Inhalation of H_2_ may be a safe and effective therapy for CHF, and may be applied to patients with CHF. The clinic trail is ongoing. Besides, it is possible that H_2_ may protect the failing heart against progression through direct or indirect mitochondrion pathways, and our relevant studies are also ongoing. For instance, H_2_ may inhibit the generation of adenosine triphosphate (ATP) from mitochondrion (data not shown), which has been confirmed in PC12 cells (Ohsawa et al., 2007).

Continuous damage from oxidative stress is an important mechanism that facilitates CHF. We summarize that molecular hydrogen as a safe antioxidant could mitigate progression of CHF via inhibiting apoptosis modulated by p53. Therefore, we propose that inhalation of H_2_ as a safe, convenient and inexpensive therapy may serve as a new antioxidant in the prevention and treatment of CHF in clinic in the future.

## Author Contributions

JC, WY, and JL conceived and designed the study. JC, TZ, YB, WZ, JxY, ZF, ZY, QX, LZ, WL, and JmY performed the experiments. JC, XH, and LZ organized the database. JC, YG, and HY performed the statistical analysis. JC and ZL drafted and revised the work. All authors contributed to manuscript revision, read and approved the submitted version.

## Conflict of Interest Statement

The authors declare that the research was conducted in the absence of any commercial or financial relationships that could be construed as a potential conflict of interest.
